# Cu_2_O nanoparticles for the degradation of methyl parathion

**DOI:** 10.3762/bjnano.11.137

**Published:** 2020-10-12

**Authors:** Juan Rizo, David Díaz, Benito Reyes-Trejo, M Josefina Arellano-Jiménez

**Affiliations:** 1 Facultad de Química, Universidad Nacional Autónoma de México, Coyoacán 04510, México; 2 Laboratorio de Productos Naturales, Área de Química, Universidad Autónoma Chapingo, Texcoco 56230, México; 3 Material Science and Engineering, The University of Texas at Dallas, Texas 75080, United States

**Keywords:** copper(I) oxide (Cu_2_O), Cu_2_O nanoparticles, degradation, methyl parathion, surface basicity

## Abstract

Methyl parathion (MP) is one of the most neurotoxic pesticides. An inexpensive and reliable one-step degradation method of MP was achieved through an aqueous suspension of copper(I) oxide nanoparticles (NPs). Three different NPs sizes (16, 29 and 45 nm), determined with X-ray diffraction (XRD) and high-resolution transmission electron microscopy (HRTEM), were synthesized using a modified Benedict’s reagent. ^1^H nuclear magnetic resonance (NMR) results show that the hydrolytic degradation of MP leads to the formation of 4-nitrophenol (4-NPh) as the main product. While the P=S bond of MP becomes P=O, confirmed by ^31^P NMR. Although Cu_2_O is a widely known photocatalyst, the degradation of methyl parathion was associated to the surface basicity of Cu_2_O NPs. Indirect evidence for the basicity of Cu_2_O NPs was achieved through UV–vis absorption of 4-NPh. Likewise, it was shown that the surface basicity increases with decreasing nanoparticle size. The presence of CuCO_3_ on the surface of Cu_2_O, identified using X-ray photoelectron spectroscopy (XPS), passivates its surface and consequently diminishes the degradation of MP.

## Introduction

Organophosphorus pesticides (OPPs) are one of many kinds of pesticides that have attracted some attention mainly due to their neurotoxic effect [1–3]. The primary mechanism of action of OPPs is that they are effective inhibitors of acetylcholinesterase through the interaction with serine inside the nucleophilic active site of the enzyme to form a phosphorylated enzyme derivative, which is more resistant to subsequent hydrolysis than the normal acetylated derivative. Therefore, the inhibition is essentially irreversible [2]. Inhibition of acetylcholinesterase leads to an accumulation of the neurotransmitter. This, in turn, causes seizures and respiratory failure, which are the main causes of death [3]. *O*,*O*-Dimethyl *O*-(4-nitrophenyl) phosphorothioate, most commonly known as methyl parathion (MP), is among the most acutely toxic pesticides used in agriculture [4–6]. MP includes other risks for human health, such as the induction of changes in tertiary villi of the placenta of women exposed to this OPP [7].

It has been estimated that in the year 2020 about 153,000 metric tons of OPPs will be used worldwide [8]. In Mexico, roughly 5,732 metric tons of MP are mainly used annually for the production of beans, cabbage, soy, wheat, lettuce, and tomatoes [5–6], despite the fact that MP is a forbidden pesticide by the United Nations Rotterdam Convention. Due to the large volumes of MP used in agriculture, thousands of metric tons, and because MP is highly neurotoxic, there has been extensive studies about the degradation of MP using different materials [9–26]. All studies about the degradation of MP can more or less be classified as biotic, photocatalytic, or chemical degradation [27]. In aqueous media, chemical degradation of MP can result in either oxidation, isomerization or hydrolysis as some authors have suggested [11–14,28]. Oxidation of MP leads to the formation of methyl paraoxon, which is much more toxic than MP. Isomerization also leads to the formation of other phosphorothioates that are acetylcholinesterase inhibitors. Therefore, hydrolysis is the desired route of degradation of MP. Strictly speaking all degradations of MP are chemical. What is meant by biotic degradation is that bacteria are used for the degradation of MP, whereas photocatalytic degradation needs photons in the form of UV light and chemical degradation utilizes chemical species, such as copper(I) oxide (Cu_2_O) NPs in this work.

Cu_2_O is widely known for its photocatalytic activity [29–33]. However, there are scarce studies of its applications regarding its basicity. In this work, copper(I) oxide NPs of different sizes were synthesized using a modified Benedict’s reaction. They were used for the first time in the chemical degradation of MP. The advantages of using Cu_2_O are that it is an inexpensive, abundant, moderately stable, and reliable source of material for the degradation of MP. It is well known that nanoparticles have the advantage of a relatively high surface area. We have used this advantage to increase the basicity of Cu_2_O in the form of surface hydroxy groups (OH). This also decreased the degradation time while increasing the degradation efficacy. Our results suggest that the surface basicity of Cu_2_O NPs leads to degradation of MP without the need of other chemical substances or the use of photocatalysts that generate free radicals. The presence of free radicals is undesired since there is a rising consensus on the damage that these reactive species, formed during the photocatalytic reactions, cause to cell membranes by peroxidation of the polyunsaturated phospholipids [34]. This leads to the subsequent loss of activity that relies on an intact membrane, and ultimately to the death of organisms. While this may be an advantage for water disinfection, it is a great disadvantage for the removal of OPPs in natural waters. Thus, Cu_2_O NPs are an excellent substance for the degradation of MP.

## Experimental

### Reagents

The chemical reagents used for the synthesis of Cu_2_O NPs were: copper(II) sulfate pentahydrate (CuSO_4_·5H_2_O; J.T.Baker, 99.9%), sodium citrate dihydrate (Na_3_C_6_H_5_O_7_·2H_2_O; Aldrich, ≥99%), Glucose (C_6_H_12_O_6_; Aldrich, ≥99.5%), sodium carbonate (Na_2_CO_3_; J.T.Baker, 99.9%), sodium hydroxide (NaOH, 1 M), dimethyl sulfoxide (DMSO; J.T.Baker, 99.9%), methyl parathion (C_8_H_10_NO_5_PS; Aldrich, ≥95%), and deionized water.

### Preparation of Cu_2_O NPs

For the preparation of Cu_2_O nanoparticles Benedict’s reagent was used [35], with the variation of a water/dimethyl sulfoxide (DMSO) solvent mixture in order to obtain different NPs sizes. The modified Benedict’s reagent was prepared as follows: In 50 mL of distilled water, 1.257 g (5 × 10^−3^ mol) of CuSO_4_·5H_2_O and 2.941 g (10 × 10^−3^ mol) of Na_3_C_6_H_5_O_7_·2H_2_O were dissolved. Then, 1.06 g (10 × 10^−3^ mol) of Na_2_CO_3_ were added and, lastly, 0.901 g (5 × 10^−3^ mol) of C_6_H_12_O_6_ were added. The 50 mL solution was then diluted to 200 mL with a mixture of H_2_O and DMSO and heated at 80 °C for 30 min under constant stirring. The end of the reaction can be noted by the precipitation of Cu_2_O. It is important to point out that cupric sulfate and sodium citrate form a cupper–citrate complex noted by an intense blue color. Sodium carbonate was added in order to maintain an alkaline pH value because glucose is the reducing agent. Alkaline pH values favor the open-chain form of glucose also known as the aldehyde form, and the aldehyde group makes glucose a reducing sugar. It is essential to point out that different mixtures of H_2_O and DMSO are used in order to obtain NPs of different sizes. When a 70% H_2_O and 30% DMSO (v/v) mixture is used for diluting the solution to 200 mL, a red precipitate of Cu_2_O NPs of approximately 45 nm is obtained. When the solution is 50% H_2_O and 50% DMSO (v/v), an orange precipitate of Cu_2_O NPs of approximately 29 nm is obtained. When it is diluted with 40% H_2_O and 60% DMSO (v/v), a yellow precipitate of Cu_2_O NPs of approximately 16 nm is obtained. All the Cu_2_O NPs obtained were rinsed with 40 mL of distilled water five times, and once with ethanol, to remove DMSO, salts or sugar that might have been absorbed by the Cu_2_O NPs. The general chemical reaction involved can be simplified as follows:

[1] $$\begin{array}{c} {\left[\text{Cu}\left(\text{citrate}\right){\left(\text{OH}\right)}_{2}\right]}_{\left(\text{aq}\right)}+{\text{C}}_{5}{\text{H}}_{11}{\text{O}}_{5}{\text{CHO}}_{\left(\text{aq}\right)}\\ \to {\text{Cu}}_{2}{\text{O}}_{\left(\text{s}\right)}+{\text{C}}_{5}{\text{H}}_{11}{\text{O}}_{5}{\text{COOH}}_{\left(\text{aq}\right)} \end{array}$$

It is important to note that the reaction in Equation 1 is not balanced in order to point out that a 1:1 mole ratio between the copper complex and glucose is needed. Glucose loses an electron while copper gains an electron. Also, the DMSO/H_2_O mixture is only used for the synthesis of Cu_2_O NPs with different sizes, it is not used in the degradation of MP.

### Methyl parathion degradation

The degradation of MP was achieved in deionized water by reacting MP with Cu_2_O NPs in a 1:5 molar ratio. This was carried out using 250 mL of a 1.5 × 10^−4^ M aqueous solution of MP (3.75 × 10^−5^ mol) containing 26.8 mg of Cu_2_O NPs (1.87 × 10^−4^ mol). First the MP solution is prepared by dissolving 9.4 mg of MP in 250 mL water. Then, 26.8 mg of Cu_2_O NPs is added to the solution. Since the Cu_2_O NPs do not dissolve in the parathion solution, the NPs were dispersed by sonicating for 90 s. Also, a constant stirring was maintained throughout the degradation of MP with Cu_2_O NPs. Table 1 summarizes the dispersion conditions for the Cu_2_O NPs of different size. The concentration of Cu_2_O was calculated by diving the amount of substance of Cu_2_O by the volume of the dispersion and does not represent the concentration of NPs because they are made up of different amounts of Cu_2_O. When the reactions were over, the NPs were separated by centrifugation. This was done also before each UV–vis absorption and NMR measurement. Degradation of MP with bulk Cu_2_O was also tested, giving similar results. Likewise, degradation experiments in the darkness were also performed giving identical results to those under daylight thus photocatalytic degradation was ruled out.

**Table 1:** Reaction conditions for the degradation of methyl parathion (MP). All experiments were carried out in a 250 mL volumetric flask under constant stirring in order to maintain dispersion of the NPs.

NPs size	Dispersion Medium	MP concentration	Cu_2_O dispersion concentration

16 nm	H_2_O	1.5 × 10^−4^ M	7.5 × 10^−4^ M
29 nm	H_2_O	1.5 × 10^−4^ M	7.5 × 10^−4^ M
45 nm	H_2_O	1.5 × 10^−4^ M	7.5 × 10^−4^ M

### Instrumentation

UV–vis electronic absorption spectra were acquired on an Ocean Optics CHEM-2000 spectrophotometer equipped with a double-way optic fiber coupled to a PC. The powder X-ray diffraction (XRD) patterns were collected with a Bruker D2 Phaser diffractometer equipped with a conventional X-ray tube (Cu Kα radiation, 30 kV, 10 mA) and the LYNXEYE one-dimensional detector. A primary divergence slit module width of 1 mm, a step width of 0.01407°, and 0.5 s time per step were used. Qualitative analysis was performed with the DiffracPlus Eva software package (Bruker AXS, Germany) using the PDF-2 database. High-resolution transmission electron microscopy (HRTEM) images were obtained in a JEOL 2010F microscope operating at 200 kV. The ^31^P and ^1^H NMR spectra were recorded on an Agilent 400 MR DD2 spectrometer (Santa Clara, CA, USA) operating at 161 MHz for ^31^P and 400 MHz for ^1^H. The ^31^P and ^1^H chemical shifts were measured in deuterated chloroform (CDCl_3_) or water (D_2_O) relative to tetramethylsilane (TMS) for ^1^H and 85% phosphoric acid (H_3_PO_4_) for ^31^P as internal standards. Typical conditions for the proton spectrum were as follows: pulse width of 45°, acquisition time of 2.5 s, FT size of 32 K and digital resolution of 0.3 Hz per point. Typical conditions for the phosphorus spectra were as follows: pulse width of 45°, acquisition time of 0.813 s, FT size of 65 K and digital resolution of 0.5 Hz per point. The number of scans varied from 512 to 4,096 per spectrum. X-ray photoelectron spectroscopy (XPS) were measured in a ESCA/SAM Perkin-Elmer model 560, using an Al Kα source with a 400 μm spot diameter and a HSA of 50 eV pass energy.

## Results and Discussion

### Characterization of Cu_2_O NPs with powder XRD and HRTEM

**Figure 1:**
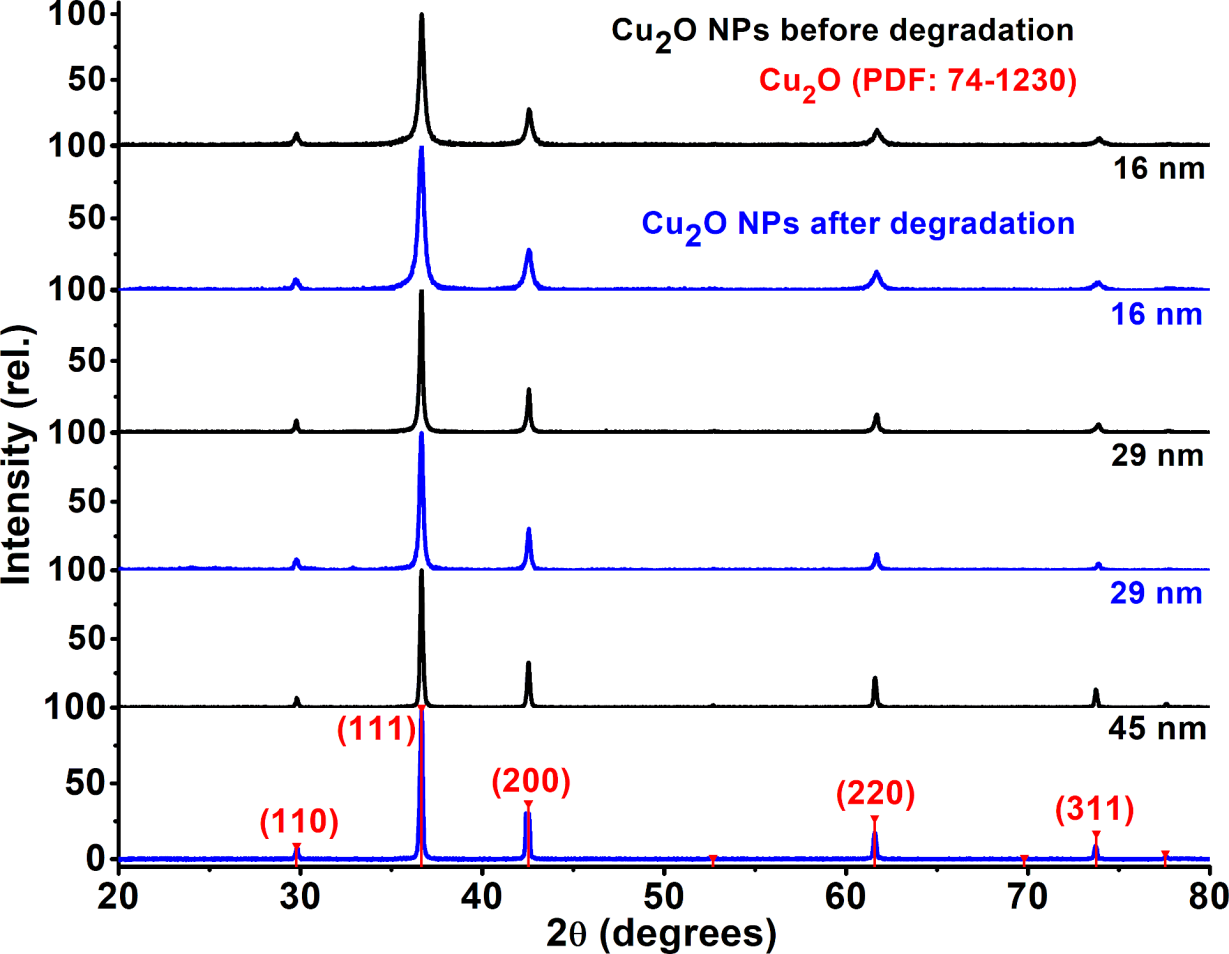
Powder XRD of the Cu_2_O NPs used before (black) and after (blue) the degradation of MP. The red numbers in parentheses indicate Miller indices obtained from powder diffraction file (PDF) 74-1230.

The structural and morphological characterization of Cu_2_O NPs was carried out using powder X-ray diffraction and high-resolution transmission electron microscopy. Copper(I) oxide is practically insoluble in water (*K*_sp_ = 2 × 10^−15^ @ 25 °C). Since the NPs remain in the powder form throughout the entire degradation, XRD is a very useful technique for the characterization of Cu_2_O. Figure 1 shows the powder XRD of the Cu_2_O NPs before and after the degradation of MP. As far as the sensitivity of this technique, the NPs are essentially inert since they do not oxidize in the MP solution. In both cases the XRD results are consistent with the powder diffraction file: PDF #74-1230, which corresponds to cubic crystals of Cu_2_O.

**Figure 2:**
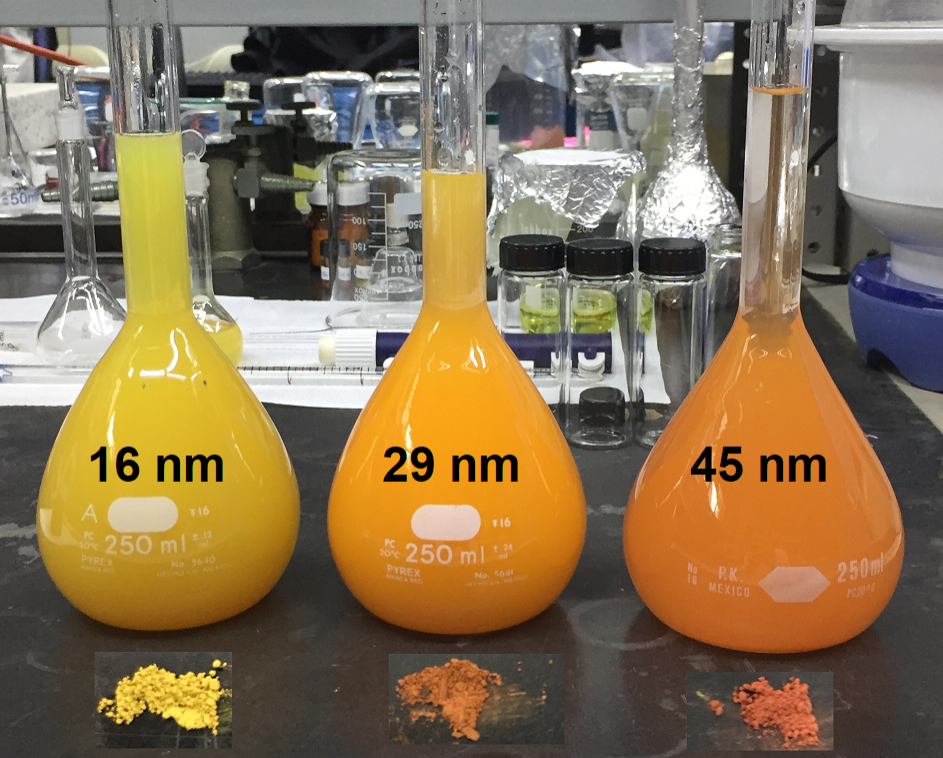
Color powders for the three different sizes as well as their color in aqueous dispersions with a concentration of 7.5 × 10^−4^ M.

XRD diffractograms show a broadening of the peaks with decreasing nanoparticle size, this is best explained by the small crystallite size of the NPs. An approximate size of the NPs can be calculated through measurements of this broadening [36]. Using the Scherrer equation, the three NPs sizes obtained were 16 ± 3 nm (yellow powder), 29 ± 3 nm (orange powder), and 45 ± 9 nm (bright red powder). The colored powders can be seen in Figure 2, as well as their color in aqueous dispersion. It is important to mention that there is no evidence in XRD for the presence of CuO or CuCO_3_, although these compouds are observed in XPS.

**Figure 3:**
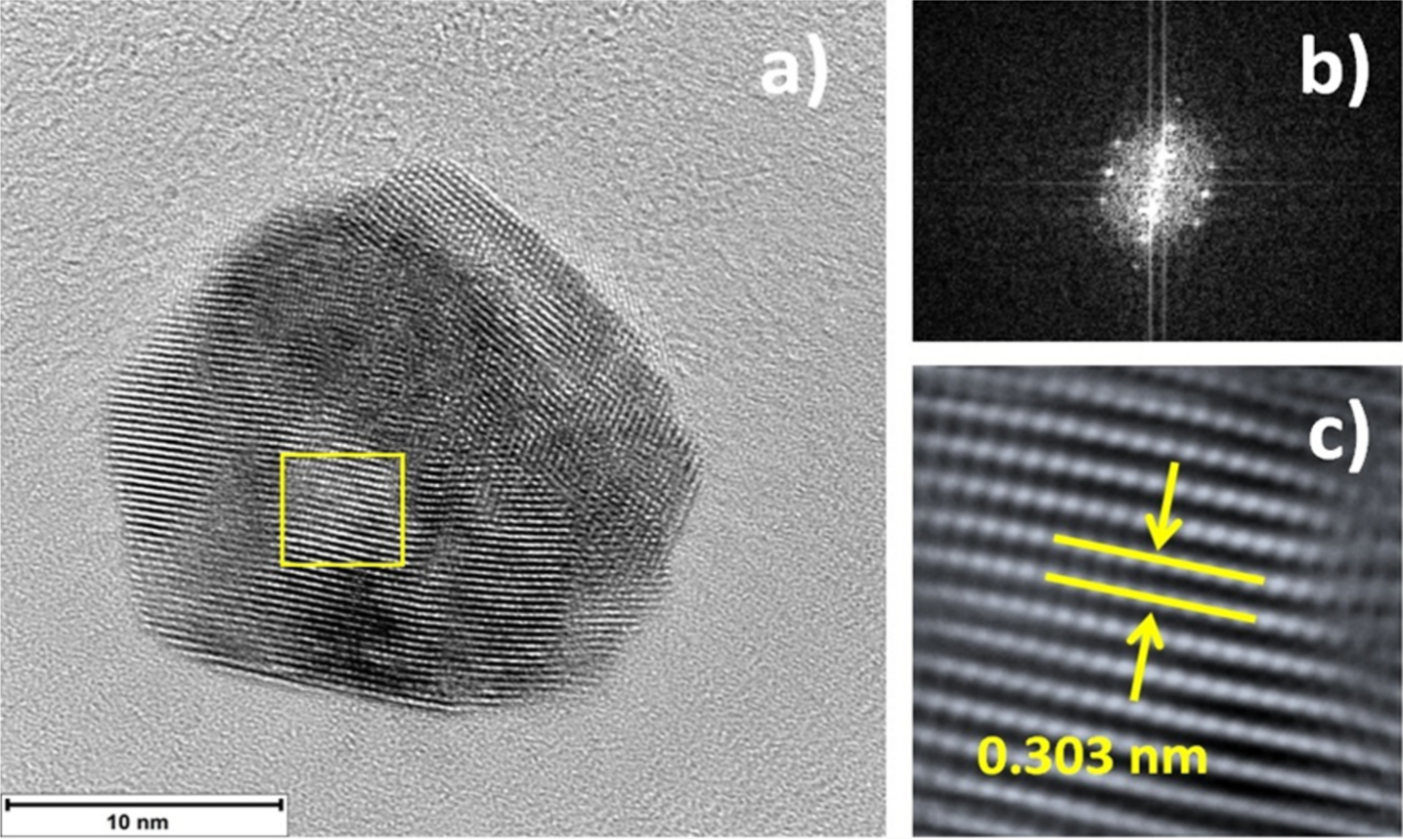
a) HRTEM image of typical Cu_2_O NP used in degradation of MP. Particle average size in the range of 16 ± 3 nm. b) FFT and c) processed image of the area marked in yellow square in (a). The interplanar distance match the (110) plane of Cu_2_O when compared to the powder diffraction file (PDF) 74-1230 used in XRD.

Figure 3a shows the HRTEM image of a typical Cu_2_O nanoparticle of 16 nm, used in the degradation of MP. Figure 3b shows the FFT from the area marked with a yellow square in Figure 3a. The processed image in Figure 3c was obtained from the same area. Interplanar distances corresponded to the (211) and (110) planes of Cu_2_O, Figure 3c shows only the (110) plane for clarity. One interesting line of research can be the dependence (if any) of different Cu_2_O planes on the degradation of MP. Experiments regarding this line of research are in progress.

### Degradation study of MP using NMR

**Figure 4:**
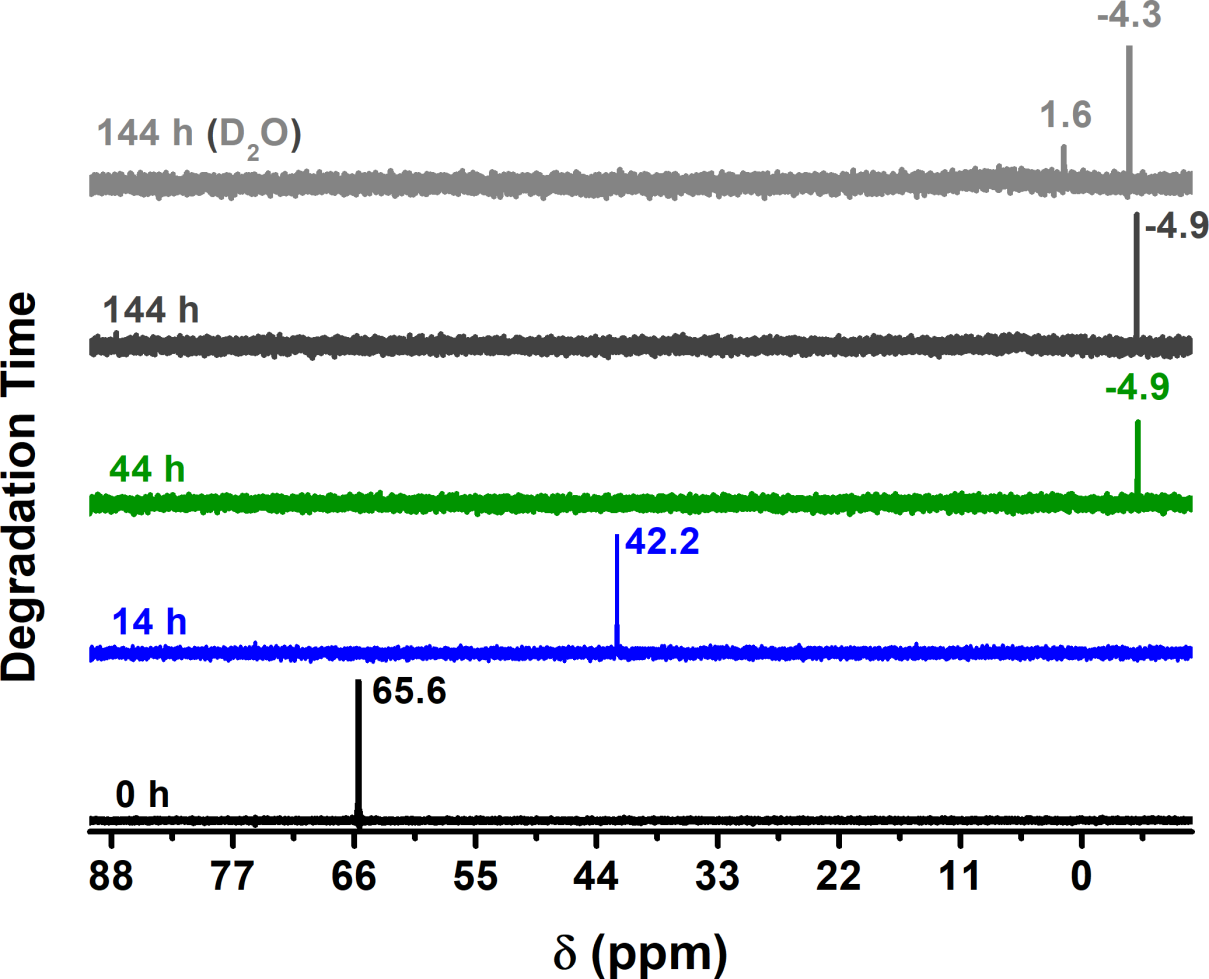
^31^P NMR, using CDCl_3_ as solvent, of the degradation products obtained after 14, 44, and 144 h of degradation time with Cu_2_O NPs of 29 nm; last spectrum taken in D_2_O.

**Figure 5:**
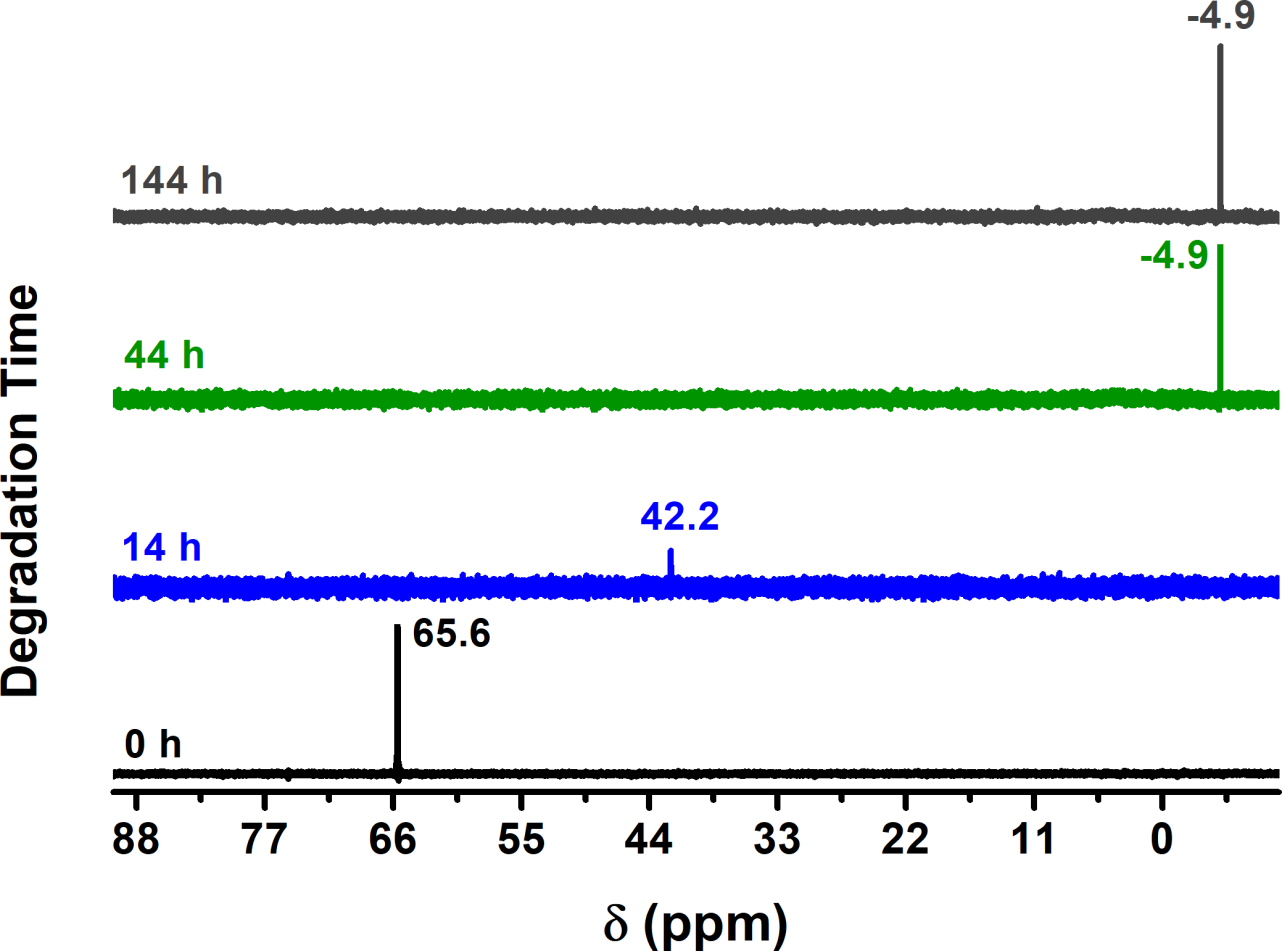
^31^P NMR, using CDCl_3_ as solvent, of the degradation products obtained after 14, 44, and 144 h of degradation time with Cu_2_O NPs of 16 nm.

**Scheme 1:**
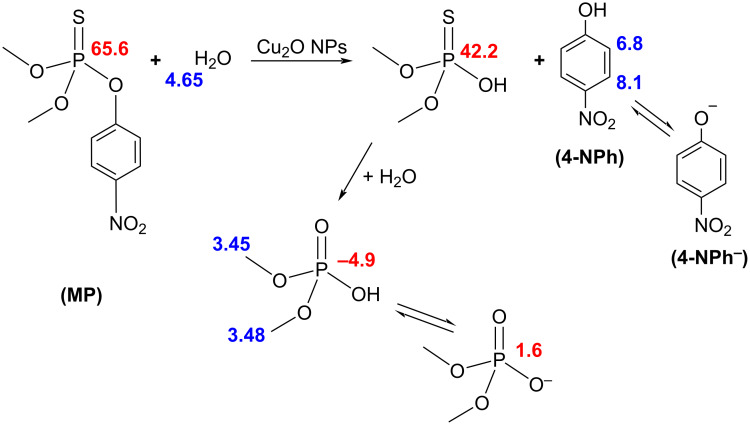
Observed degradation pathway of MP with Cu_2_O NPs in aqueous solution to form 4-nitrophenol and dimethyl phosphate. Numbers in blue indicate the observed ^1^H NMR chemical shifts (δ) in parts per million (ppm) while the numbers in red correspond to ^31^P. 4-Nitrophenol can further be reduced to 4-aminophenol as other authors have suggested [43].

^31^P NMR is used as a characterization technique for the degradation of MP [11,21,37–40]. Figure 4 and Figure 5 show the ^31^P NMR spectra of the products obtained after 14, 44 and 144 h of degradation time using Cu_2_O NPs. In all cases, the spectrum for 0 h corresponds to pure MP with a chemical shift of 65.6 ppm as reported elsewhere in the literature [38–41]. The ^31^P NMR spectra of Figure 4 show the results obtained when Cu_2_O NPs with an approximate size of 29 nm were used for the degradation. In this case, the final product formed is dimethyl hydrogen phosphate with a chemical shift of −4.9 ppm in deuterated chloroform (CDCl_3_). Similarly, the chemical shift of 42.2 ppm is that of dimethyl phosphorothioate (P=S) [40], which then hydrolyzes after 44 h to form dimethyl hydrogen phosphate (P=O) and the NMR peak at −4.9 ppm developes [42]. The intensity of the chemical shift is relatively low due to the low solubility in CDCl_3_, but when D_2_O is used the intensity increases under the same experimental conditions and there are two chemical shifts: one at −4.3 ppm, which corresponds to protonated form (acid), and the one at 1.6 ppm belonging to the deprotonated form (anion), both of which are in equilibrium [42] (see below Scheme 1 for their corresponding molecular structural formulas).

Figure 5 shows the ^31^P NMR results when Cu_2_O NPs of 16 nm are used in the degradation of MP. The results are identical to those of Figure 4. Hence, the same products were generated. NMR results for the degradation of MP when Cu_2_O NPs of 45 nm were used are not presented since they are similar to those of Figure 4 and Figure 5. One important aspect of the ^31^P NMR results in Figure 4 and Figure 5 is the absence of the chemical shift for MP (65.6 ppm) after 14 and 44 h of degradation time, this does not mean that all the MP was degraded within that reaction time but instead it is attributed to the technique used for dehydration (lyophilization) before the NMR spectra were obtained. In other words, during the lyophilization process when water is removed by lowering the temperature and pressure followed by an increase in temperature so that water is removed by sublimation and consequently methyl parathion is also removed and therefore absent in the NMR spectra.

**Figure 6:**
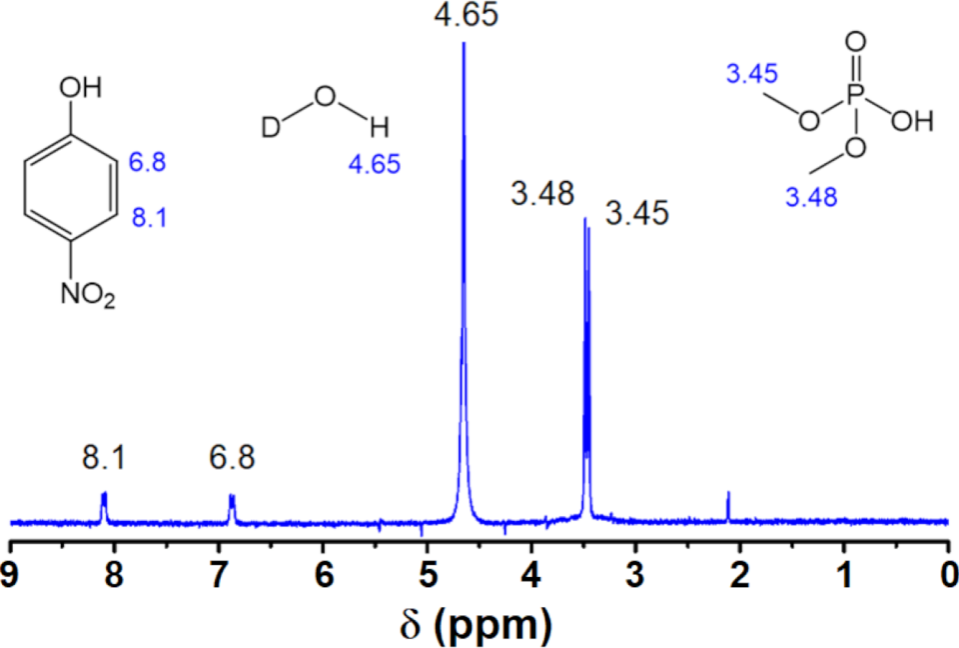
^1^H NMR, using D_2_O as solvent, of the degradation products obtained after 144 h of degradation time with Cu_2_O NPs of 29 nm.

Figure 6 is the ^1^H NMR spectrum of the degradation products obtained with Cu_2_O NPs of 29 nm using D_2_O as solvent. The chemical shifts at 6.8 and 8.1 ppm belong to the coupled protons (d, *J* = 9 Hz) of 4-nitrophenol. The peaks at 3.45 and 3.48 ppm are the methyl groups of phosphate, which show coupling to phosphorous, and the peak at 4.65 ppm is due to the HDO produced by the deuterium interchange with the hydroxyl group of 4-nitrophenol. D_2_O was used as solvent for ^1^H NMR instead, of CDCl_3_ like in ^31^P NMR, because both 4-nitrophenol and dimethyl hydrogen phosphate (products) are more soluble in water than in chloroform but methyl parathion (reactant) is more soluble in CDCl_3_. The presence of 4-nitriphenol as reaction product has one important implication. It suggests the hydrolysis reaction takes place through a nucleophilic substitution at the phosphorous atom (S_N_^2^@P), in which hydroxy groups are the nucleophile as Liu et al. have reported [14], and not at aliphatic or aromatic carbon atoms (S_N_^2^@C) [14,39]. Furthermore, Cu_2_O NPs play an important role in the degradation of MP since hydroxy groups are found on its surface (see XPS results below). These surface hydroxy groups can either be directly involved in the S_N_^2^@P mechanism or they can polarize the oxygen–hydrogen bonds of the water molecules and thus facilitate the hydrolysis of MP. Further research regarding the exact mechanism for the degradation of MP on the surface of Cu_2_O NPs is in progress. Scheme 1 shows the observed degradation pathway considering all the NMR results obtained.

### Degradation study of MP using UV–vis spectroscopy

**Figure 7:**
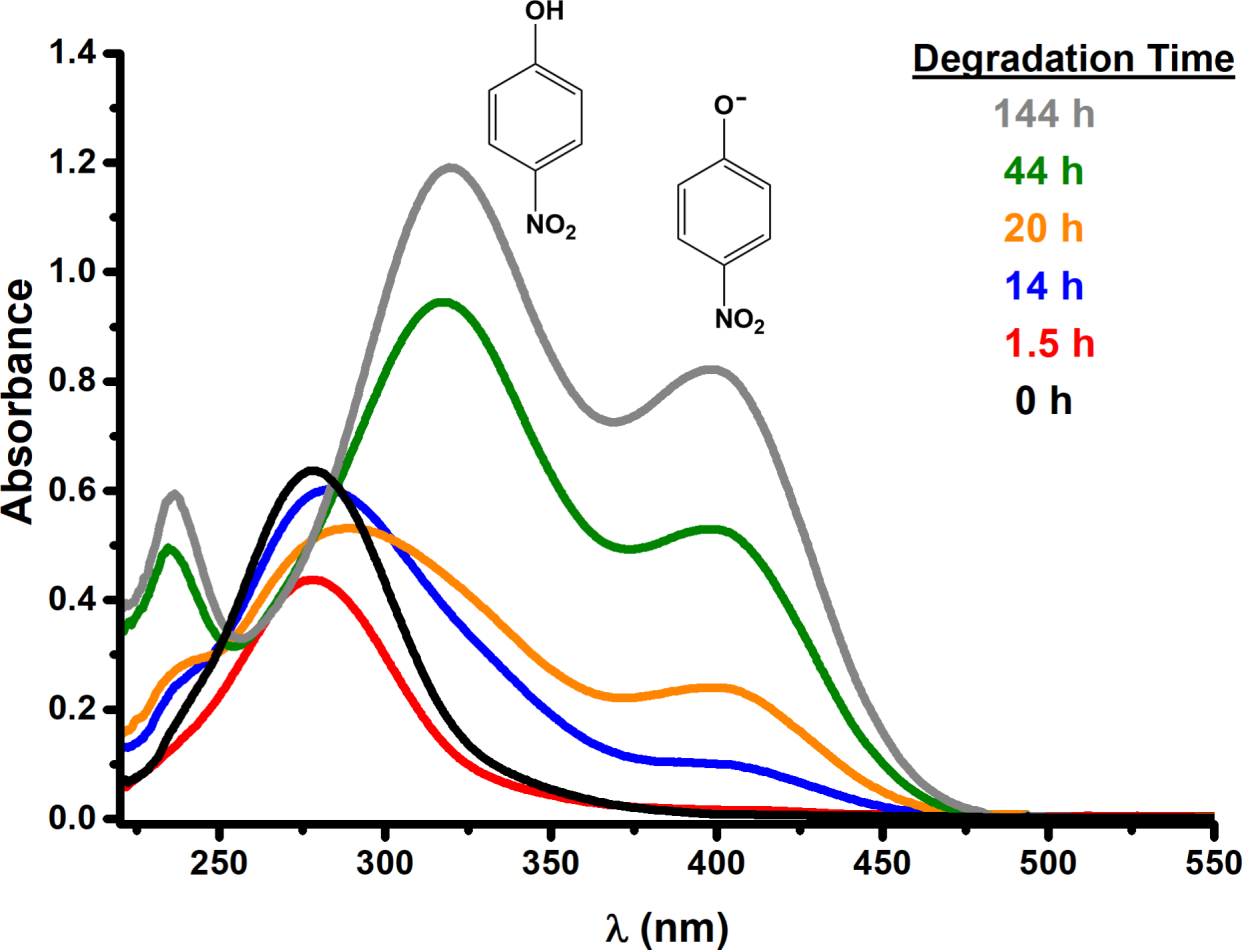
UV–vis spectra of the degradation of MP using the 45 nm Cu_2_O NPs.

**Figure 8:**
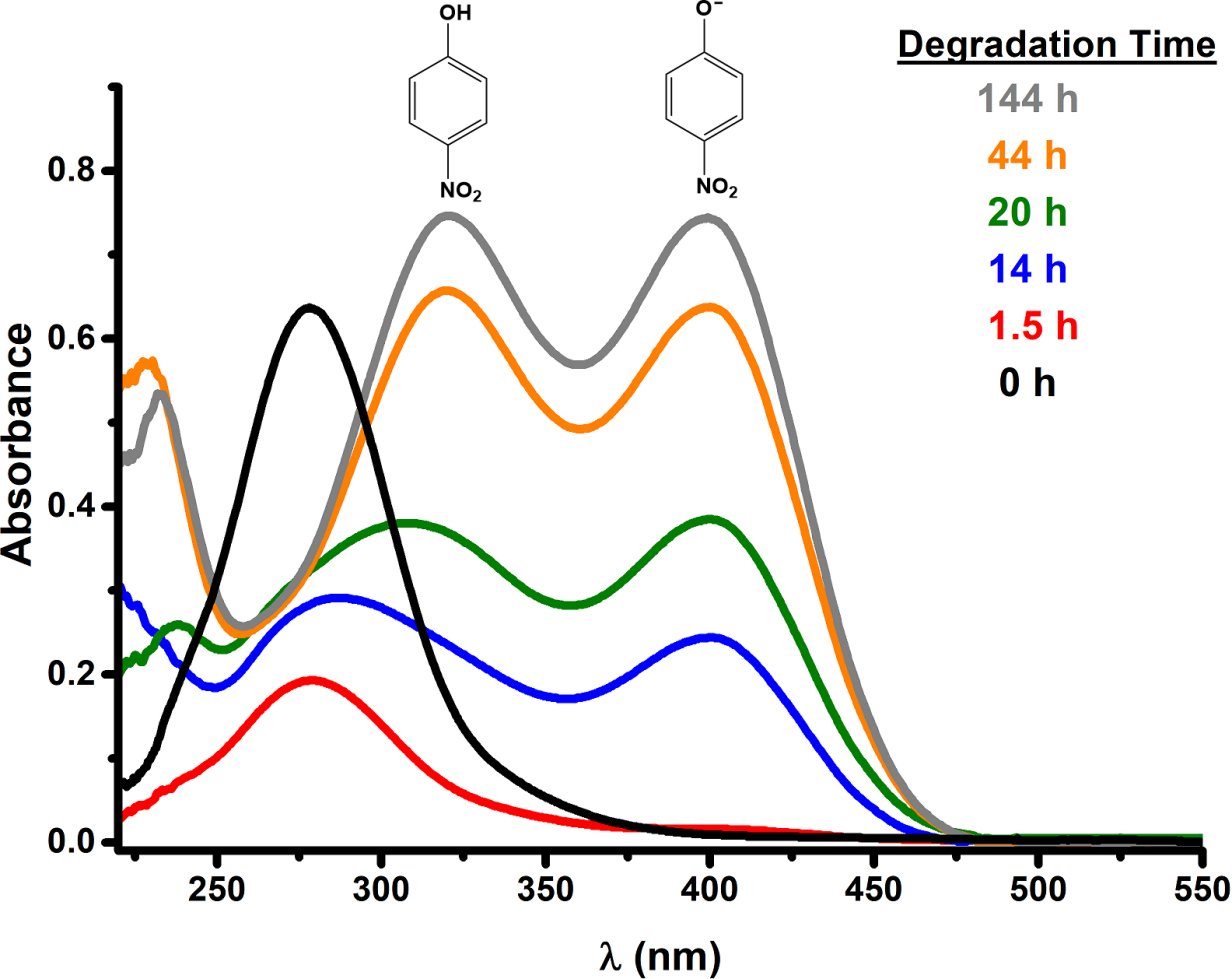
UV–vis spectra of the degradation of MP using the 16 nm Cu_2_O NPs.

NMR results indicate that one of the degradation products obtained is 4-nitrophenol (4-NPh). The presence of 4-NPh makes quantification of the degradation much easier because 4-NPh absorbs light in the UV–vis range. Hence absorption spectroscopy was used along with the Beer–Lambert law [44]. The molar absorptivity coefficients were determined to be 10080 M^−1^·cm^−1^ (λ = 320 nm) for 4-nitrophenol and 17632 M^−1^·cm^−1^ (λ = 400 nm) for 4-nitrophenolate (4-NPh^−^), these results are similar to those reported in the literature [39,44]. Figure 7 and Figure 8 are the UV–visible spectra for the degradation of MP with different NPs sizes. Degradation times are indicated with different colors. The band around 280 nm corresponds to MP, the band around 320 nm to 4-NPh, and the band around 400 nm to 4-NPh^−^.

The absorption band at 400 nm gives a bright yellow color, which can be used to visually determine that the degradation is taking place. The yellow color intensifies as the degradation time increases. It can be seen that the intensity of the band at 280 nm starts to decrease with increasing degradation time while the intensity of the bands at 320 and 400 nm increases. These results are expected but the relative intensities between the 4-nitrophenol (320 nm) and 4-nitrophenolate (400 nm) are different depending on the NP size (Figure 7 and Figure 8). A smaller nanoparticle size leads to a higher concentration of 4-nitrophenolate. 4-NPh is in equilibrium with 4-NPh^−^ (Scheme 1). According to Le Chatelier’s principle, the equilibrium favors the formation of 4-NPh^−^ at basic pH values. This means that the NPs with size of 16 nm have a stronger basicity because they generate a higher concentration of 4-NPh^−^. In other words, the chemical basicity of Cu_2_O increases with decreasing NPs size.

**Figure 9:**
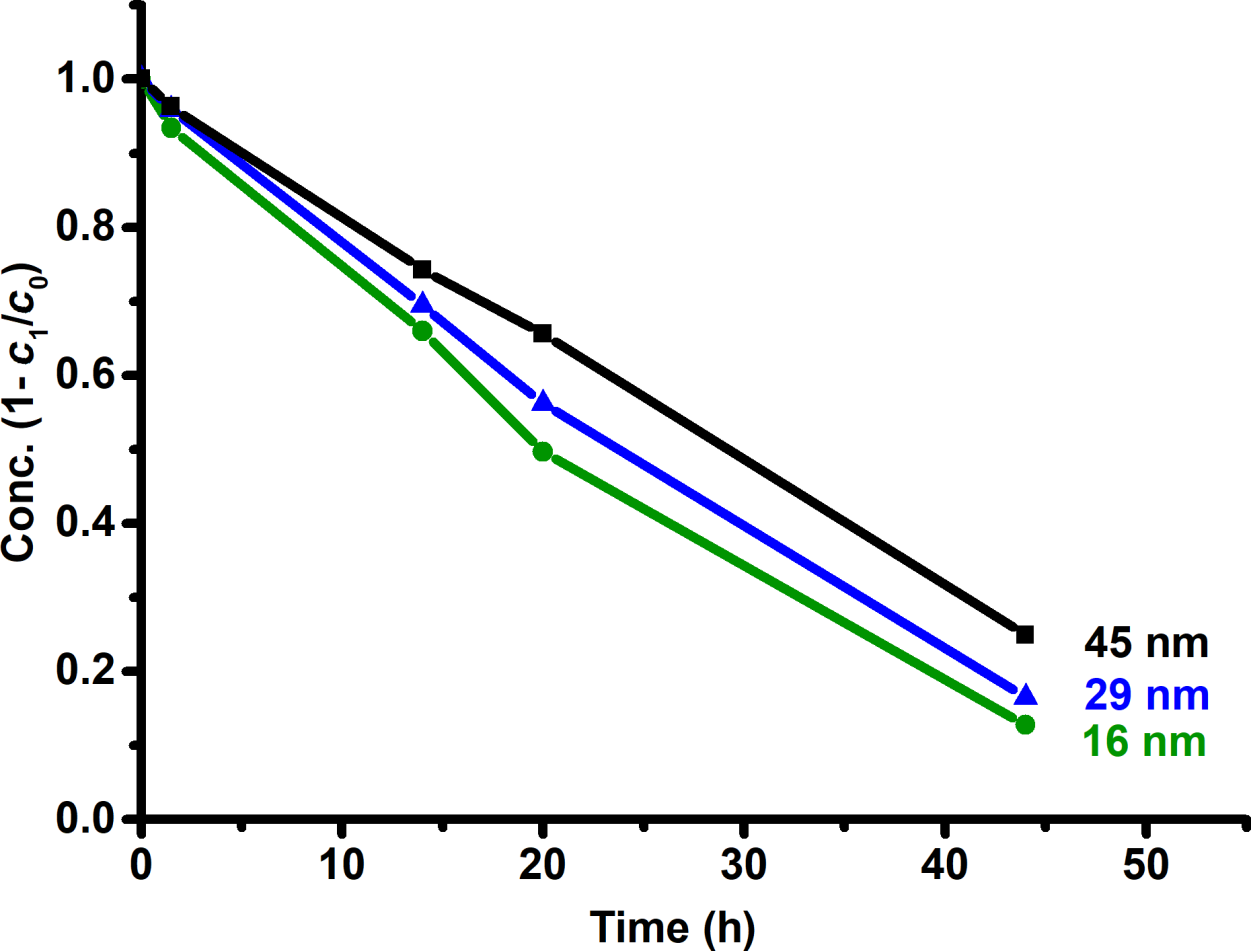
Degradation of methyl parathion with different sizes of Cu_2_O NPs.

This last result is best explained with Pearson’s concept of basicity [45–46], low oxidation number metal oxides are alkaline in aqueous medium. Thus, Cu_2_O is a basic metal oxide. Similarly, as the NP size decreases the surface-to-volume ratio increases. A higher surface area implies a higher amount of hydroxy groups [47–48] and, hence, a higher basicity. MP degradation can be further extended to different metal oxides as others have already reported on the literature [15–16,22–26,49]. One major difference in this work is the absence of free radicals since the degradation is not photocatalytic. This absence of free radicals makes Cu_2_O NPs a reliable source for the degradation of MP in natural waters. Figure 9 shows the degradation of MP at different reaction times for all three nanoparticle sizes.

Since water is a reactant as well as the solvent, it is prudent to assume a reaction of pseudo first-order kinetics because water is in excess with respect to MP. The degradation of methyl parathion in water is accomplished to about 87% after 44 h of reaction time using 16 nm Cu_2_O NPs, to about 84% with 29 nm Cu_2_O NPs, and to about 75% with 45 nm Cu_2_O NPs. The measurement at 144 h showed a complete degradation (100%), although this was an excess time and does not correspond to the actual time for a complete degradation.

### Surface study of Cu_2_O NPs using XPS

It is worth noting that there is a small difference in degradation percentage between Cu_2_O NPs of 16 nm (87%) and those of 29 nm (84%), but a larger difference between the Cu_2_O NPs of 29 nm (84%) and those of 45 nm (75%). The degradation percentage should increase with a reduction in NP size. However, the almost inexistent difference (3%) between the 16 nm and 29 nm NPs suggest the influence of other factors. In order to further study this small difference in degradation percentage between 16 nm and 29 nm NPs, X-ray photoelectron spectroscopy (XPS) analyses were carried out.

**Figure 10:**
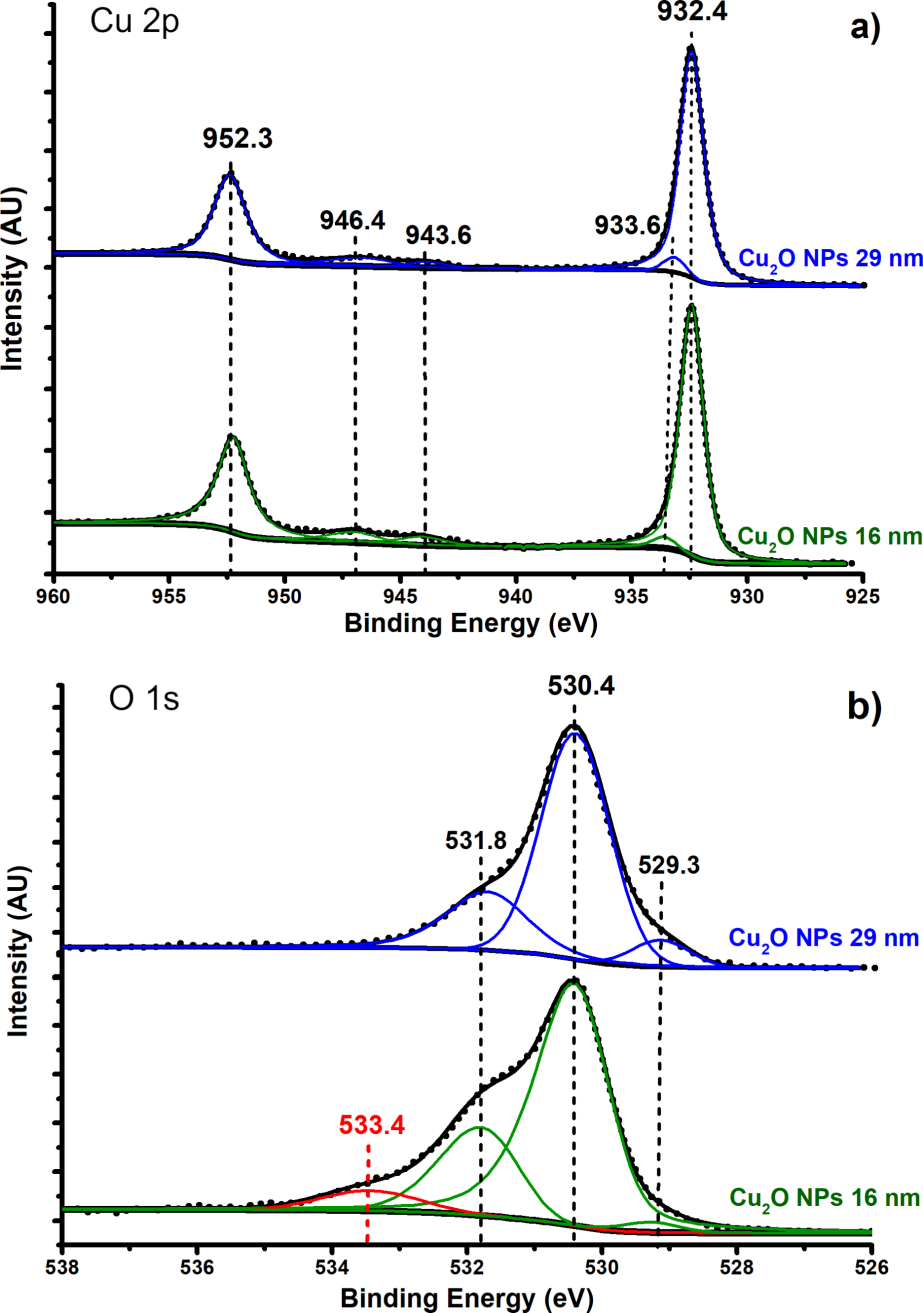
XPS spectra for Cu_2_O NPs of 16 nm and 29 nm size, a) Cu 2p, b) O 1s.

Figure 10 shows the Cu 2p (Figure 10a) and O 1s (Figure 10b) XPS spectra obtained for Cu_2_O NPs of 16 nm and 29 nm after the degradation. In Figure 10a, the peak at 932.4 eV was fixed for all samples so that it matches with the Cu 2p_3/2_ of Cu_2_O reported in the literature [50–51]. The peak at 952.3 eV is the corresponding spin–orbit splitting (2p_1/2_) of Cu_2_O. Also, in Figure 10a there is a small peak at 933.6 eV that is assigned to Cu 2p_3/2_ of CuO. This last peak was placed in the fitted spectra because there are two peaks at 943.6 eV and 946.4 eV that have been widely accepted as shake-up satellites of Cu 2p and thus implicate the presence of CuO. In Figure 10b, the presence of CuO is more noticeable in the O 1s XPS spectra with a peak at 529.3 eV [50–52]. The presence of CuO on the Cu_2_O samples has two possible important implications: First, its presence suggests a passivation of the Cu_2_O surface because the degradation of methyl parathion does not occur when CuO is used instead of Cu_2_O in the dispersion medium. Second, CuO could play an important role in the degradation mechanism by anchoring MP molecules on the surface of Cu_2_O through a coordinated bond between Cu^2+^ of CuO and the sulfur atom of MP [53]. Further research regarding this topic is in progress. Also, we were not able to quantify the amount of CuO in both samples (16 and 29 nm). However, they both contain CuO. Hence this is not the most probable cause for the small degradation difference between 16 nm and 29 nm Cu_2_O NPs. The peak at 530.4 eV corresponds to lattice O 1s of Cu_2_O whereas the peak at 531.8 eV is assigned to surface O 1s (in the form of OH) in Cu_2_O [50,52]. The presence of hydroxy groups at the surface of Cu_2_O NPs should enhance the MP degradation due to the nucleophilic substitution observed in NMR (Scheme 1). Regarding the peaks at 531.8 eV in Figure 10b, the 16 nm NPs have a higher relative intensity than the 29 nm NPs. Nonetheless, the amount of surface OH groups seen in XPS is not representative of the reaction conditions because more of these groups should form on the surface of the Cu_2_O NPs when they are placed in water [47–48].

One important difference in the O 1s XPS spectra between 16 nm and 29 nm NPs is the peak at 533.4 eV, which corresponds to CuCO_3_ [52]. This carbonate species is also observed in the FTIR spectra. The carbonate species is formed only on the 16 nm NPs and it is the most probable cause for the small degradation difference between the 16 nm and the 29 nm NPs. Even though the 16 nm NPs have a larger surface-to-volume ratio than the 29 nm NPs, the presence of CuO and CuCO_3_ on the surface of 16 nm Cu_2_O NPs limits the amount of OH groups that can form on its surface. Consequently, both 16 nm and 29 nm NPs have a similar active surface size and the degradation percentage of MP is similar between 16 nm Cu_2_O (87%) and 29 nm Cu_2_O (83%). In order to avoid oxidation of Cu_2_O NPs, reduced graphene oxide (rGO) can be used as a support [54]. Finally, XPS spectra of S 2p and P 2p were also obtained before and after the degradation. The results show the absence of both elements on the surface of Cu_2_O. Hence, these results are not included here.

One significant observation is that Cu_2_O NPs enhance the degradation of MP via hydroxy groups on its surface. We found other variants of the MP degradation using bulk Cu_2_O or oxidized pennies. The main reason for using Cu_2_O NPs is that this type of nanostructures greatly decreases degradation time and enhances the degradation percentage. For example, the MP degradation using oxidized pennies requires about 8 days while bulk Cu_2_O requires 6 days for 32% degradation. Cu_2_O NPs of 16 nm yield a degradation of 87% in 44 h. Lastly, further studies are required to see if Cu_2_O NPs can be used for the degradation of other organophosphorus pesticides of similar structure to that of methyl parathion, that is, phosphate triesters such as fenitrothion or diazinon. Research regarding this matter is in progress.

## Conclusion

Cu_2_O nanoparticles were used for the first time in the hydrolytic degradation of methyl parathion, the most neurotoxic organophosphate pesticide used to date. The surface basicity of copper(I) oxide in the form of hydroxy groups, evidenced by XPS, promotes a nucleophilic substitution at the phosphorous atom of methyl parathion forming 4-nitrophenol, dimethyl phosphorothioate, and dimethyl hydrogen phosphate as the primary degradation products, identified through ^1^H and ^31^P NMR.

Likewise, indirect evidence for the formation of hydroxide ions is achieved by applying Le Chatelier’s principle to the chemical equilibrium of 4-nitrophenol. Similarly, the relative concentrations of 4-nitrophenol and 4-nitrophenolate imply that the surface basicity of Cu_2_O NPs increases with decreasing NPs size.

An 87% degradation of MP was achieved in 44 h when Cu_2_O NPs of 16 nm were used in aqueous medium, while 84% degradation was achieved with 29 nm NPs and 75% degradation was achieved when 45 nm NPs were used. Also, the use of other chemical species or light are not required for the hydrolytic degradation of MP with Cu_2_O NPs.

Finally, the presence of CuCO_3_ on the surface of Cu_2_O, shown by Cu 2p and O 1s XPS spectra, passivate its surface and consequently makes the degradation of MP less effective.
